# The Impact of a Workforce Mental Health Program on Employer Medical Plan Spend: An Application of Cost Efficiency Measurement for Mental Health Care

**DOI:** 10.1089/pop.2022.0240

**Published:** 2023-02-14

**Authors:** Todor Penev, Shelley Zhao, Jennifer L. Lee, Connie E. Chen, Leanne Metcalfe, Ronald J. Ozminkowski

**Affiliations:** ^1^ Health Solutions, Aon plc, Atlanta, Georgia, USA.; ^2^ Health Solutions, Aon plc, Portland, Oregon, USA.; ^3^ Lyra Health, Burlingame, California, USA.; ^4^ Emory University School of Medicine, Atlanta, Georgia, USA.; ^5^ Health Solutions, Aon plc, Austin, Texas, USA.; ^6^ Health Solutions, Aon plc, Saline, Michigan, USA.

**Keywords:** mental health, workforce mental health, cost-effectiveness, employee assistance program, therapy, health care claims

## Abstract

Mental health issues often result in significant impairment and financial challenges, both at home and in the workplace. Solutions vary widely in their usage and cost-effectiveness. This study presents an analysis of medical and prescription drug spending and utilization data for the employees of 4 companies who were eligible for an evidence-based workforce mental health program (WMHP). A variation of coarsened exact matching paired WMHP users to nonusers, based on demographics, location, and medical factors. Individuals included 2791 pairs of members whose medical claims were incurred in 2018 and 3883 pairs with claims in 2019. Using a cost efficiency measurement process, mean cost and utilization per person per year (PPPY) were compared. WMHP users had lower medical (−$2295 in 2018; −$2304 in 2019) and prescription drug spending (−$295 in 2018; −$312 in 2019). Over half of the cost reduction (−$1252 in 2018; −$1211 in 2019) resulted from shifting therapy services from the medical benefit to WMHP. WMHP users attended about 12 sessions PPPY, whereas the comparison group of nonusers attended about 7 mental health office visits PPPY under the medical benefit. WMHP users had more mental health-related visits in both years, but had fewer visits on the medical plan, and fewer emergency department visits for mental health than comparison group members. These results provide evidence that high-quality, evidence-based mental health services can reduce total expenditures and change utilization patterns. Evidence-based WMHP may represent a prudent investment for employers in providing mental health care to employees.

## Introduction

Mental health challenges present significant quality-of-life and financial burdens for employees and employers. Somewhere between 30% and 50% of adults experience mental illness over their lifetimes,^1,2^ resulting in more than $200 billion annually in health care utilization and lost work productivity.^3,4^ Mental health concerns remain among the top contributors of disability worldwide.^5^ Within the workplace, acute and chronic mental health conditions result in more days of work lost and a higher likelihood of working while impaired,^6,7^ as well as higher employee turnover.^8^ Providing appropriate mental health care to those in the workforce could help to alleviate these challenges and reduce suffering.^9^

For many employees, mental health care services are provided through employer-sponsored health insurance. Despite mandates of parity in the insurance coverage of physical and mental health services in the United States, disparities between mental and physical health care coverage and quality still exist.^10^ One significant barrier to obtaining mental health care is the significant financial burden that results, as individuals with mental health conditions experience high cost sharing and out-of-network spending responsibilities.^11,12^

In addition, many individuals have difficulty accessing mental health services in a timely manner due to mental health care provider shortages,^13,14^ logistical challenges presented by outdated or inaccurate insurance directories,^15^ and even discrimination within the search for care.^16^ If someone can find a provider within their health insurance plan network, they still may not have found a provider who performs evidence-based treatment (EBT), which is the gold standard^17^ for improvement in clinical outcomes and value-based care. Barriers to accessing high-quality care remain high,^14^ whereas the need for mental health services has increased, nearly doubling in the past 30 years.^2^

Inadequately treated mental health conditions, whether caused by access challenges or nonevidence-based practice, also impact physical functioning and disease-related spending. Even among individuals with similar levels of chronic physical illness, living with a mental health condition results in greater health care resource utilization and costs, due to more hospitalizations, longer hospital stays, and more emergency department (ED) visits.^18^ These increased costs are shared by individuals and employers. According to the Kaiser Family Foundation, the average employer-sponsored health care premium in 2021 ranged from roughly $7700 for individuals to about $22,000 for families, representing a 22% increase over the past 5 years and a 47% increase over the past 10 years.^19^

To alleviate the burden of workforce mental health challenges, employers continue to search for cost-effective or cost-savings solutions for employees. Workforce mental health programs (WMHPs) are comprehensive mental health and wellness programs targeted at employed populations that aim to fill this critical need and gap in the health care system. These programs aim to provide a focused investment in mental health, with the potential to shift ineffective spending for mental health concerns away from the medical insurance plan.

The broad value of a focused investment on employees' mental health has been described in the literature, with many traditional employee-assistance programs (EAPs) showing a positive return on investment for employers.^3,20,21^ Cost-savings and cost-effectiveness vary greatly, however, by type of intervention (eg, EBT or not evidence based), employer characteristics (eg, size, corporation type), and treatment focus (eg, diagnoses covered by the EAP).^22^ Providing evidence-based treatment for mental health conditions in general has been demonstrated as cost-effective,^23,24^ including preliminary evidence for cost-effectiveness for EBT for depression in the workplace.^20^

Challenges in methodology and analysis, however, limit the generalizability of these findings to WMHP. For example, many studies on EAPs and WMHP unreliably project savings from a small number of users over a brief period of time to an entire population over years,^7,25^ whereas a preferred method would be to examine actual usage of the WMHP and health care expenditure data over an extended period of time.

Despite high need and a general perception that these programs can be effective, the quality of care provided by WMHPs varies greatly, with many traditional EAPs mirroring the challenges of finding care through a health insurance plan (eg, troubles with access, session limits, providers who are not offering EBT).^21,26,27^ WMHPs providing EBT in psychotherapy show high rates of symptom reduction and clinical improvements for those who use their services.^28,29^ Services using exclusively EBT also demonstrate reductions in employee turnover, compared with individuals accessing usual care in the community.^8^

Although clinical improvements and impacts on workplace outcomes have been demonstrated by WMHP using EBT, more research is needed to demonstrate the financial value of providing EBT through WMHP using real-world data from health care claims. To that end, the objective of this study was to examine differences in medical, pharmacy, and mental health care utilization and expenditures between individuals who utilize a WMHP providing EBT and individuals who do not. The hypothesis is that individuals who utilize the evidence-based WMHP will have lower medical and pharmacy costs, for both mental health- and nonmental health-related claims.

## Evidence-Based Workforce Mental Health Solution

Workforce mental health services were offered to eligible individuals through Lyra Health's WMHP, partnering with Lyra Clinical Associates, to self-referring individuals and their dependents, as a mental health benefit from their employers. Individuals seeking care accessed providers through an online portal that paired them with options for providers who provided EBT for their specific clinical needs. Appointments could be scheduled with a provider immediately through a connected calendar. All costs of care for these services were covered by the employer, with no cost sharing for the employee.

Providers are selected after rigorous evaluation and supervising processes to evaluate the quality of therapy and usage of EBTs, hiring only 4%–9% of applicants. All interventionists receive ongoing supervision, continuing education, and evaluation for performance in achieving clinical outcomes. The evidence-based services providing this WMHP are described in detail elsewhere.^29^

## Study Design, Data Sources, and Methods

### Study design

This study involved a third-party retrospective analysis conducted by Aon plc using deidentified data from 4 WMHP employer clients who together had over 50,000 eligible members. These data report eligibility for insurance coverage and insurance claims for health care services. Data were initially provided to Lyra Health using a fully executed business associate agreement. Deidentified data were transferred to Aon for independent review and analysis, with consent from employer clients. The use of deidentified data falls outside the regulatory definition of research involving human subjects, obviating the need for institutional review board (IRB) approval.

However, significant efforts were made to maintain data and individual confidentiality, consistent with data privacy and security safeguards described in the Health Insurance Portability and Accountability Act and the Health Information Technology for Economic and Clinical Health Act.

The study used eligibility and health care claims data to compare medical and prescription drug spending in 2018 and 2019 between 2 groups of people: those who utilized the WMHP services (WMHP users) and those who did not (the comparison group, also referred to as nonusers). Within these 2 groups, matched pairs were generated based on over 40 measures of geography, demographics, and medical and mental health comorbidities (Tables 1 and 2). The analysis utilized Aon's member-level cost efficiency measurement process, which is described in more detail below.

**Table 1.** Sample Characteristics Before and After Matching for 2018

	2018 Before matching				2018 After matching			
	WMHP users group	Comparison group			WMHP users group	Comparison group		s
Variable	n = 2990	n = 40,027	Standardized differences	*P*	n = 2791	n = 2791	Standardized differences	*P*
	Mean or %	Mean or %			Mean or %	Mean or %		
Age	31.08	27.29	0.311	<0.001	30.81	31.44	−0.078	0.004
Adult (%)	92.8%	67.9%	0.659	<0.001	92.8%	89.8%	0.104	<0.001
Female (%)	52.7%	42.6%	0.203	<0.001	52.6%	50.6%	0.040	0.141
MSA 1	31.3%	25.8%	0.122	<0.001	31.9%	29.6%	0.051	0.056
MSA 2	15.2%	15.9%	−0.018	0.334	15.4%	17.1%	−0.045	0.095
MSA 3	12.0%	9.0%	0.096	<0.001	11.7%	12.4%	−0.021	0.434
MSA 4	5.0%	2.9%	0.110	<0.001	5.1%	4.9%	0.008	0.758
MSA 5	5.2%	2.6%	0.130	<0.001	4.8%	3.7%	0.059	0.028
All other MSAs	31.3%	43.7%	−0.259	<0.001	31.1%	32.5%	−0.030	0.262
Mood disorders (%)	36.6%	3.1%	0.927	<0.001	36.0%	36.0%	0.000	1.000
Anxiety (%)	64.2%	4.0%	1.646	<0.001	64.3%	54.2%	0.208	<0.001
Adjustment disorders (%)	42.3%	1.9%	1.115	<0.001	42.5%	24.3%	0.394	<0.001
Attention-deficit disorders (%)	5.0%	1.7%	0.182	<0.001	4.7%	4.7%	0.000	1.000
Alcohol/substance disorders (%)	2.1%	0.5%	0.144	<0.001	1.5%	1.5%	0.000	1.000
Asthma(%)	2.7%	2.4%	0.019	0.345	2.1%	2.1%	0.000	1.000
Blood cell disease (%)	0.4%	0.3%	0.019	0.365	0.1%	0.1%	0.000	1.000
Cancer (%)	0.7%	0.5%	0.016	0.438	0.1%	0.1%	0.000	1.000
Secondary cancer (%)	0.0%	0.1%	−0.035	0.330	0.0%	0.0%	0.000	1.000
Cardiovascular (%)	2.8%	2.6%	0.012	0.545	1.7%	1.7%	0.000	1.000
Lower back/disk diseases (%)	16.9%	8.4%	0.257	<0.001	14.7%	14.7%	0.000	1.000
Diabetes (%)	0.7%	0.9%	−0.021	0.352	0.5%	0.5%	0.000	1.000
Diabetes with complications (%)	0.8%	0.8%	0.002	1.000	0.4%	0.4%	0.000	1.000
Esophageal/upper GI diseases (%)	2.2%	1.4%	0.055	0.002	1.2%	1.2%	0.000	1.000
Hypertension (%)	1.6%	1.8%	−0.009	0.681	1.5%	1.2%	0.028	0.352
Metabolic disorders (%)	2.8%	2.4%	0.023	0.230	1.7%	1.7%	0.000	1.000
Migraine/headache (%)	4.8%	2.5%	0.128	<0.001	3.6%	3.6%	0.000	1.000
Multiple sclerosis (%)	0.1%	0.1%	0.006	1.000	0.0%	0.0%	0.000	1.000
Neurological disorders (%)	4.4%	2.9%	0.078	<0.001	2.8%	2.8%	0.000	1.000
Osteoarthritis (%)	0.7%	0.6%	0.014	0.500	0.1%	0.1%	0.000	1.000
Pregnancy and labor (%)	3.4%	3.0%	0.020	0.301	3.1%	3.1%	0.000	1.000
Rheumatoid arthritis (%)	0.3%	0.2%	0.012	0.628	0.1%	0.1%	0.000	1.000

GI, gastrointestinal; MSA, metropolitan statistical area; WMHP, workforce mental health program.

**Table 2.** Sample Characteristics Before and After Matching for 2019

	2019 Before matching				2019 After matching			
	WMHP users group	Comparison group			WMHP users group	Comparison group		
Variable	n = 4182	n = 41,437			n = 3883	n = 3883		
	Mean or %	Mean or %	Standardized differences	*P*	Mean or %	Mean or %	Standardized differences	*P*
Age	31.36	27.21	0.342	<0.001	31.11	31.81	−0.088	<0.001
Adult (%)	93.6%	67.6%	0.698	<0.001	93.6%	91.5%	0.081	<0.001
Female (%)	56.0%	43.0%	0.261	<0.001	55.7%	53.4%	0.046	0.047
MSA 1	30.9%	26.6%	0.094	<0.001	31.4%	28.8%	0.056	0.013
MSA 2	14.0%	14.9%	−0.024	0.138	14.2%	16.1%	−0.054	0.018
MSA 3	10.2%	8.5%	0.059	<0.001	9.7%	11.0%	−0.043	0.057
MSA 4	5.9%	3.3%	0.123	<0.001	5.9%	5.0%	0.041	0.072
MSA 5	5.6%	2.5%	0.155	<0.001	5.2%	3.2%	0.096	<0.001
All other MSAs	33.5%	44.2%	−0.221	<0.001	33.6%	35.8%	−0.046	0.043
Mood disorders (%)	33.9%	3.4%	0.849	<0.001	33.8%	33.8%	0.000	1.000
Anxiety (%)	67.0%	4.4%	1.726	<0.001	66.6%	54.2%	0.254	<0.001
Adjustment disorders (%)	35.4%	2.0%	0.949	<0.001	35.6%	25.5%	0.219	<0.001
Attention-deficit disorders (%)	5.0%	1.7%	0.183	<0.001	4.0%	4.0%	0.000	1.000
Alcohol/substance disorders (%)	1.8%	0.5%	0.120	<0.001	1.1%	1.1%	0.000	1.000
Asthma (%)	2.7%	2.4%	0.020	0.237	1.9%	1.9%	0.000	1.000
Blood cell disease (%)	0.4%	0.4%	0.005	0.859	0.1%	0.1%	0.000	1.000
Cancer (%)	0.6%	0.6%	−0.005	0.823	0.2%	0.2%	0.000	1.000
Secondary cancer (%)	0.1%	0.0%	0.025	0.126	0.0%	0.0%	0.000	1.000
Cardiovascular (%)	3.5%	2.6%	0.050	0.001	2.2%	2.2%	0.000	1.000
Lower back/disk diseases (%)	18.6%	9.2%	0.276	<0.001	17.2%	17.2%	0.000	1.000
Diabetes (%)	0.9%	0.9%	−0.004	0.865	0.5%	0.5%	0.000	1.000
Diabetes with complications (%)	0.9%	0.7%	0.026	0.108	0.4%	0.4%	0.000	1.000
Esophageal/upper GI diseases (%)	2.7%	1.4%	0.093	<0.001	1.6%	1.6%	0.000	1.000
Hypertension (%)	2.3%	1.8%	0.032	0.044	2.0%	2.1%	−0.009	0.749
Metabolic disorders (%)	3.5%	2.5%	0.054	<0.001	2.4%	2.4%	0.000	1.000
Migraine/headache (%)	5.3%	2.6%	0.140	<0.001	3.9%	3.9%	0.000	1.000
Multiple sclerosis (%)	0.2%	0.1%	0.027	0.081	0.0%	0.0%	0.000	1.000
Neurological disorders (%)	4.7%	3.1%	0.082	<0.001	2.9%	2.9%	0.000	1.000
Osteoarthritis (%)	0.6%	0.6%	−0.004	0.882	0.3%	0.3%	0.000	1.000
Pregnancy and labor (%)	3.6%	3.0%	0.030	0.061	3.3%	3.3%	0.000	1.000
Rheumatoid arthritis (%)	0.2%	0.2%	−0.008	0.747	0.0%	0.0%	0.000	1.000

GI, gastrointestinal; MSA, metropolitan statistical area; WMHP, workforce mental health program.

### Study participants

Lyra Health's WMHP services were offered to all eligible employees and dependents by their employers. Engagement with WMHP services was voluntary and self-selected by the employee. For group assignment, individuals assigned to the WMHP users group included those who had at least 1 visit with the WMHP providers within a given year. Individuals in the comparison group were matched to WMHP users as mentioned above, to adjust for many nonprogram-related factors that might influence the cost of care. Individuals in both groups were able to use their medical benefit plans to engage in mental health care outside of the WMHP, if desired, and all such claims were included in the analyses.

**FIG. 1.**
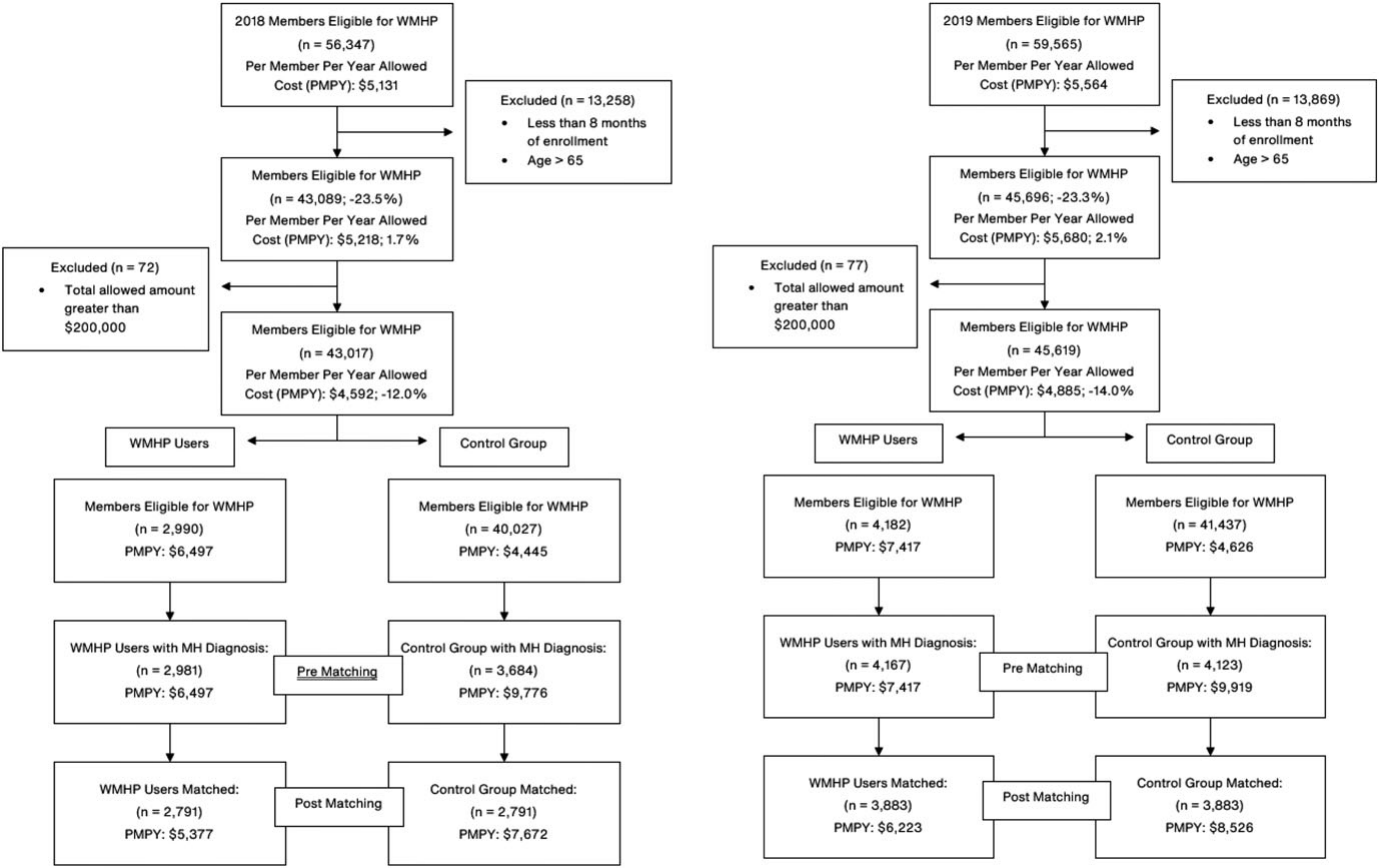
Participant flow diagram of inclusion and exclusion for 2018 and 2019. MH, mental health; WMHP, workforce mental health program.

To be included in the analysis, individuals must have been under the age of 65 years with at least 8 months of access to the WMHP in the given year. Members whose cumulative medical spend over $200,000 per year (medical and pharmacy combined) were excluded (comprising <0.1% of members) from the main analysis, as their utilization patterns were statistical outliers that did not represent the overall population. Additional sensitivity analyses were conducted to evaluate the impact of different outlier cutoffs of $50,000 and $100,000 on the primary outcome of total medical, mental health, and prescription spending. Figure 1 shows a visualization of inclusion and exclusion criteria used for the study.

### Data sources

#### Eligibility data

The 4 employers who contributed data for this study worked within the technology, manufacturing, consumer goods, and transportation industries. As of 2019, the companies included in the analysis employed an average of 6000 employees. These employers provided Lyra Health with monthly data files, sharing up-to-date information on over 50,000 covered individuals who were eligible for health care services during 2018 or 2019, including demographic and geographic information. These covered individuals lived in 38 states around the country.

#### Geographic data and social determinants of health

Geographic information was summarized by incorporating the Area Deprivation Index (ADI)^30,31^ into the analyses. The ADI was calculated at the 5-digit ZIP code level, as a proxy of an individual's socioeconomic status (SES) and social determinants of health (SDOH). SES and SDOH factors related to education levels, income/employment rates, housing values, and household characteristics in a geographic area often influence cost and utilization analyses, so it was important to adjust for the impact of these factors.

#### Health insurance claims data

Detailed medical and pharmacy claims were collected from 2018 and 2019 on all WMHP users and nonusers. At least 3 months elapsed after the end of the year before claims data were obtained, to allow for processing. Pharmacy rebate data were not available and were not incorporated. Mental health-related spending was identified through primary diagnostic codes in the ICD-10 F-series, at the claim-line level. For WMHP users, mental health conditions were identified through both WMHP sessions and health plan benefit claims. Mental health service use among the comparison group was identified through medical and pharmaceutical claims only.

### Participant and nonparticipant matching procedures

WMHP users were coded as such in this study based upon their use of at least 1 WMHP service. WMHP providers submitted diagnostic impressions that were then mapped to ICD-10 diagnosis codes by Lyra Health clinicians. A detailed mapping from diagnostic impression to ICD-10 diagnosis codes is included in the Appendix Table A1.

To apply the Aon cost efficiency measurement process, WMHP users were matched with a comparison group of nonusers, composed of eligible members at the same employers with closely matched geography, demographics, and medical and mental health comorbidities, for the same time periods. A derivation of coarsened exact matching^32^ was used to match cohorts. As originally described by Iacus et al, coarsened exact matching first involves dividing members into meaningful categories selected for each matching factor of interest.

Specifically, members were first divided into age groups, gender categories, geographic area categories, and according to the existence or not of several medical conditions, as noted below. Then, all members of the treatment and comparison groups who fell into the same categories were retained for the analysis; the rest were excluded. The Iacus et al^32^ method also suggests using case weights to account for the proportion of treatment and comparison group members who are in each factor category, then using their original data values (not the indicators of which factor categories they fell into) in the subsequent statistical analyses.

The Iacus et al method was simplified for this analysis, by avoiding the use of case weights. Age is the only continuous measure in this data set, so individuals were matched on tightly constructed age groups, which still produced a highly balanced set of WMHP users and comparison group members for analysis. More specifically, individuals were matched by gender first; then they were matched to others within ±3 years of their ages.

Individuals were then matched on presence or absence of 22 diagnosed medical conditions (Tables 1 and 2), and combinations of selected conditions. The chronic condition indicators considered for each member were based on primary (first listed) medical diagnostic codes, using the Chronic Condition Indicator and Clinical Classifications Software developed by the Agency for Healthcare Research and Quality Healthcare Cost and Utilization Project.^33^ When members had more than 1 mental health condition, a hierarchy was applied.

Specifically, when 2 or more disorders were present, members were coded according to the one that is typically thought to be more impactful in nature, resulting in greater levels of disability and incurring the most cost. Based upon the existing literature,^2,4,5^ mood disorders were anticipated to incur more costs than anxiety and adjustment disorders, which were in turn anticipated to incur more costs than attention-deficit disorders.

Next, WMHP users were matched to nonusers in the same geographic areas, when possible. First, an attempt was made to match members residing in the same metropolitan statistical area (MSA), based on a list of over 200 such areas across the United States. When a within-MSA match could not be found, members were matched at the state level. When that was not possible, members were not matched geographically, but were matched on the other factors mentioned above, searching across the country for the best demographic and condition level matches.

When no matches could be found (typically when members had a rare combination of disease and location values), individuals were removed from further analysis. For WMHP users who could have matched to more than 1 comparison group member, only 1 matching comparison group member was randomly selected for inclusion into the analyses. See Figure 1 for other inclusion and exclusion criteria and associated sample size reductions.

### Statistical analyses

After matching was complete, mean health care expenditure values were calculated for all types of service, then for individual or combinations of multiple service types based on site or type of care. Differences in expenditures between groups were also examined by age, gender, and the ADI used as a proxy for their SDOH. Mean differences in expenditures between WMHP users and comparison group members were tested for statistical significance at the 95% confidence level, using 2-sample *t*-tests, assuming unequal variances; this is consistent with the approach recommended by Iacus et al.^32^

## Results

### Matching results

Matching resulted in 2791 member pairs in 2018 and 3883 member pairs in 2019. Overall, 93.3% of all WMHP users were matched with comparison group members in 2018, and 92.9% of all WMHP users were matched in 2019. The most challenging variable to match on was geography. In 2019, of the total population, 69.2% of the WMHP users were matched to comparison group members in the same MSA, an additional 9.1% of WMHP users were matched outside their MSA but within the same state, and 14.6% of WMHP users were matched nationally.

The remaining 7.1% were not matched and dropped from the study. Similar geography matching results were observed for 2018. Select smaller MSAs were combined with adjacent large MSAs in high-density areas (eg, New York, San Francisco, Denver, and Salt Lake City) to improve local sample sizes.

Pre- and postmatching results are presented in Tables 1 and 2 to describe the samples. Matching greatly reduced differences in means and percentages between all the variables used in the matching analyses. Standardized differences for all variables except 2 were below 0.10, which is commonly taken as a benchmark for adequate balance. The 2 exceptions were adjustment disorders and anxiety, which were 40%–60% higher among WMHP users than potential comparison group members before matching, but only about 10%–20% higher after matching.

### Claims analyses

Results given in Tables 3 and 4 compare health expenditures between WMHP users and comparison group members. In both 2018 and 2019, WMHP users had significantly lower medical and pharmaceutical expenditures overall, as well as lower expenditures for mental health and nonmental health problems. In particular, WMHP users' mental health expenditures were about $1200–$1400 lower per member per year than comparison group members. This includes a significant difference in both inpatient and outpatient facility-related dollars in 2019, but not in 2018.

**Table 3.** Medical, Nonmental Health, Mental Health, and Prescription Drug Expenditures for WMHP Users and Matched Comparison Group Members for 2018 and 2019

	2018					2019				
Per participant per year spend		WMHP user (n* = *2791)	Comparison group (n* = *2791)	Difference	*P*		WMHP user (n* = *3883)	Comparison group (n* = *3883)	Difference	*P*
Medical spend		$4197	$6196	−$1999	<0.0001		$4960	$6952	−$1992	<0.0001
Medical nonmental health claims		$3663	$4397	−$734	0.0068		$4510	$5097	−$586	0.0403
Mental health spend		$534	$1799	−$1265	<0.0001		$450	$1855	−$1405	<0.0001
Facility mental health claim		$275	$289	−$13	0.8947		$166	$360	−$194	0.0021
Inpatient mental health claim		$81	$136	−$55	0.2262		$70	$174	−$104	0.0113
Outpatient mental health claim		$194	$152	$42	0.4354		$96	$186	−$89	0.0045
Professional mental health claim		$258	$1510	−$1252	<0.0001		$284	$1495	−$1211	<0.0001
Prescription drug claim		$1180	$1476	−$295	<0.0001		$1262	$1574	−$312	<0.0001
Total		$5377	$7672	−$2295	<0.0001		$6223	$8526	−$2304	<0.0001

Outlier threshold evaluation	*n*	Lyra utilizer	Control group	Difference	*P*	*n*	Lyra utilizer	Control group	Difference	*P*
Total—$50K threshold	2711	$4307	$6376	−$2069	<0.0001	3730	$4772	$6408	−$1635	<0.0001
Total—$100K threshold	2776	$5130	$7236	−$2106	<0.0001	3851	$5805	$7712	−$1907	<0.0001

Expenditure amounts in dollars represent per person per year mean spending.

WMHP, workforce mental health program.

**Table 4.** Medical and Prescription Drug Expenditures and Utilization for WMHP Users and Matched Comparison Group Members for 2018 and 2019

	2018				2019			
ED detail	WMHP utilizer (n* = *2791)	Comparison group (n* = *2791)	Difference	*P*	WMHP utilizer (n* = *3883)	Comparison group (n* = *3883)	Difference	*P*
Mental health ED	$9	$27	−$18	0.002	$10	$19	−$9	0.109
Nonmental health ED	$152	$163	−$11	0.634	$169	$209	−$40	0.063
Total ED spend	$162	$190	−$29	0.185	$179	$227	−$49	0.029
Mental health ED util/1000	5.6	20.2	−14.6	<0.001	6.7	11.5	−4.8	0.036
Nonmental health ED util/1000	115.4	136.0	−20.6	0.109	118.4	144.4	−26.0	0.018
Total ED util/1000	121.0	156.2	−35.2	0.009	125.1	155.9	−30.8	0.006
Prescription drug detail								
Generic drug	$188	$284	−$97	<0.001	$221	$296	−$75	<0.001
Brand drug	$388	$457	−$70	0.078	$313	$346	−$34	0.215
Specialty drug	$605	$734	−$130	0.352	$728	$931	−$203	0.111
Total prescription drug spend	$1180	$1476	−$296	<0.001	$1262	$1574	−$312	<0.001
Generic drug scripts/1000	3719.2	4770.8	−1051.6	<0.001	4034.6	5067.6	−1033.0	<0.001
Brand drug scripts/1000	792.1	815.2	−23.1	0.641	489.7	530.8	−41.1	0.072
Specialty drug scripts/1000	73.8	74.2	−0.4	0.970	108.3	125.8	−17.5	0.124
Total prescription drug scripts/1000	4585.1	5660.2	−1075.1	<0.001	4632.6	5724.2	−1091.6	<0.001

Expenditure amounts in dollars represent per person per year spending; “util/1,000” represents the number of visits utilized per 1000 eligible people; “Generic Drug Scripts/1000,” “Brand Drug Scripts/1,000,” “Specialty Drug Scripts/1000” and “Total Prescription Drug Scripts/1,000” represent the number of prescriptions written per 1000 eligible people.

ED, emergency department; WMHP, workforce mental health program.

The overall directionality, magnitude, and statistical significance of results were maintained in the sensitivity analyses. Using a cutoff of cumulative spending of over $50,000 per year, WMHP users still had lower total spending on average (−$2069 in 2018, *n* = 2711; −$1635 in 2019, *n* = 3730; *P'*s < 0.0001) than nonusers. Using a cutoff of cumulative spending of over $100,000 per year, WMHP users again had lower total spending on average (−$2106 in 2018, *n* = 2776; −$1907 in 2019, *n* = 3851; *P'*s < 0.0001). Given the consistent pattern of results, and the desire to account for a very high proportion of spending, the threshold of $200,000 was maintained in the results shown below.

Regarding utilization, WMHP users engaged in an average of 12.7 visits with the WMHP in 2018 and 11.9 in 2019, compared with 1.2 and 1.4 visits, respectively, on the medical plan's mental health benefit. Individuals in the comparison group who did not use the WMHP services engaged in an average of 7.4 and 6.9 visits on the medical plan's mental health benefit in 2018 and 2019, respectively. WMHP users engaged in significantly more mental health-related visits both years, with a mean difference of 6.5 more visits in 2018 (*P* < 0.001) and a mean difference of 6.4 more visits in 2019 (*P* < 0.001). WHMP users had fewer visits on the medical plan's mental health benefit than comparison group members.

Utilization of the ED (Table 5) varied by year and specific outcome. Although total claims spending for mental health-related concerns was significantly lower per member per year in 2018 for WMHP users than among comparison group members, this was not the case in 2019. Total ED claims costs were, however, lower in 2019 for WMHP users than for comparison group members, but this was not the case in 2018. For both years, ED utilization for mental health concerns per 1000 members was significantly lower for WMHP users than for comparison group members. Nonmental health ED utilization was also significantly lower for WMHP users than for comparison group members in 2019.

**Table 5.** Differences in Expenditures by Demographics and Area Deprivation Indices

	2018				2019			
	WMHP utilizer (n* = *2791)	Comparison group (n* = *2791)	Difference	*P*	WMHP utilizer (n* = *3883)	Comparison group (n* = *3883)	Difference	*P*
Male: age group in years								
1–18	$3091	$6039	−$2948	0.065	$3371	$4005	−$634	0.491
19–29	$3427	$4950	−$1523	0.003	$4232	$7896	−$3664	<0.001
30–39	$4398	$7010	−$2612	<0.001	$5524	$6865	−$1341	0.051
40–49	$7078	$8109	−$1031	0.628	$6652	$7473	−$821	0.621
50–64	$8189	$6194	$1995	0.606	$10,423	$6034	$4389	0.224
Total	$4327	$6419	−$2092	<0.001	$5178	$7101	−$1923	<0.001
Female: age group in years								
1–18	$2598	$7819	−$5221	0.006	$2980	$5878	−$2898	0.022
19–29	$4446	$6362	−$1916	0.001	$4894	$6902	−$2008	<0.001
30–39	$7756	$10,931	−$3175	0.001	$8983	$11,872	−$2889	0.002
40–49	$10,842	$10,269	$573	0.698	$9932	$12,378	−$2446	0.267
50–64	$7264	$8204	−$940	0.713	$8920	$9705	−$785	0.790
Total	$6328	$8914	−$2586	<0.001	$7057	$9775	−$2718	<0.001
Area deprivation index groupings (higher values refer to greater deprivation related to social determinants of health)								
0–20	$5727	$8383	−$2656	<0.001	$6616	$9564	−$2948	<0.001
21–40	$4813	$6469	−$1656	0.015	$6028	$7261	−$1233	0.114
41–60	$4789	$6696	−$1907	0.018	$4841	$6612	−$1771	0.034
61–80	$4070	$5732	−$1662	0.376	$5425	$5857	−$432	0.702
81–100	$6946	$4891	$2055	0.431	$5628	$3177	$2451	0.344
Total	$5377	$7672	−$2295	<0.001	$6223	$8526	−$2303	<0.001

Expenditure amounts in dollars represent per person per year mean spending.

WMHP, workforce mental health program.

WMHP users had lower overall prescription drug spending and a lower number of prescriptions filled per 1000 members than did comparison group members. By prescription subtype, this finding appeared to be driven primarily by generic drug costs and prescriptions.

When examined by age group (Table 5), all age groups showed lower health expenditures among WMHP user members than among comparison group members; differences were statistically significant among those under 40 years of age (*P* < 0.001). Differences in health care expenditures by ADI were seen only in areas with fewer deprivations related to SDOH, with WMHP users in these groups having lower expenditures.

## Discussion

Overall, lower health care claims costs were found for individuals who used Lyra Health's WMHP services providing EBT than among the comparison group members who did not use these services. WMHP users also engaged in a greater number of visits specifically for mental health concerns with the WMHP, demonstrating that accessing EBT through the WMHP was feasible and sustained for many members, a factor previous research has shown can be a significant barrier in traditional EAPs and mental health care through a medical plan.^11,21^

Lower spending related to mental health-related ED visits, as well as ED utilization rates for mental health conditions, suggests that individuals receiving services through the WMHP may also have experienced fewer mental health emergencies because of engaging in care with the WMHP. Although these results were not consistently statistically significant across years, the lack of statistical significance could be a result of sample size or lower utilization rates of the ED.

Given the increasing burden of ED visits for mental health issues in the community,^34,35^ the alleviation of this burden reflects significant societal benefit of reduced utilization of this limited resource. WMHP users also had lower prescription costs and fewer prescriptions filled and claimed during the year, despite being matched on their medical conditions. This finding is consistent with other research stating that alleviation of mental health concerns can reduce spending overall for medical concerns.^36,37^

Differences in spending by age and program usage were only found for those under the age of 40 years. Overall spending increased after age 40 years, for both the WMHP and comparison groups. For older adults, it may be that other health care expenses (eg, those due to chronic illnesses such as diabetes or cardiac issues) make it more difficult to demonstrate the financial gains of treating mental health concerns. Given the average age of 31 years in this overall sample, sample sizes in the older age groups also may be too small to detect significant differences by age group.

Regarding the SDOH analysis, differences in spending were found among WMHP and comparison group members living in areas with lower ADI scores (ie, in those areas where relatively fewer deprivations related to SDOH). This pattern could be attributed to differences in care-seeking behavior by individuals who had better access to care and greater financial resources than those who are represented in areas with greater deprivation of SDOH. Future research is needed to examine and understand patterns of WMHP usage by SDOH.

Although participant matching procedures closely adhered to established procedures for coarsened exact matching, there were some discrepancies in the distribution of mental health diagnoses before matching that were challenging to overcome. WMHP users were more likely to have adjustment disorders and to also have comorbid diagnoses (eg, both depression and anxiety) than the comparison group members. These discrepancies may also represent diagnostic challenges for individuals seeking care outside of the WMHP.

This challenge was approached using a research-informed hierarchical matching process to generate matches for people with multiple mental health conditions; however, the process had limitations. WMHP users likely had superior diagnostic data and labels before matching, due to their engagement with evidence-based mental health services that recommend diagnostic assessment and clarity for selecting the right treatment modality.^17,38^

Also, ICD-10 diagnosis codes for the WMHP were derived from expert reviews of clinical documentation in patients' medical records. Although research suggests this is a valid approach for categorizing diagnoses,^39^ diagnoses made for WMHP users may have differed in unknown ways from diagnoses that would have been made if these patients had sought care through the health plan.

A few more limitations of this study are worth noting. First, there are unmeasured factors, such as socioeconomic variables and differences in job types, even within employers, that may have impacted study outcomes. In addition, unmeasured cultural factors and engagement preferences may have influenced differential care-seeking behaviors and, therefore, the observed cost patterns. WMHP users and comparison group members pursued different forms of care for their mental health conditions. Matching reduced some of the impact of known influential factors driving their choices, however, unmeasured factors likely played a role as well.

For example, factors such as urgency for treatment or schedule flexibility may have impacted the selection of 1 service over another. Mental health care sought from some health plans may require waiting days to weeks for appointments, whereas the WHMP typically scheduled visits within 1 to 2 days of request.

Next, research suggests that stigma and privacy concerns surrounding mental health care may prevent some people from engaging with a WMHP, even when it is available.^40^ Those concerns were not measured in this study and could have played a role in service selection.

An additional factor that may impact differential treatment choices is the out-of-pocket cost of mental health care that would be obtained through the health plan. This cost may be prohibitive for some due to limited financial resources, resulting in them seeking care through the WMHP at no out-of-pocket cost. A more comprehensive study would leverage randomization to treatment type (eg, a randomized controlled trial comparing a WMHP with health plan-provided treatment) to help account for these unmeasured factors.

Next, the analyses did not account for the cost of the WMHP to employers, so a return on investment in the WMHP is not presented. However, it is important to note that there was no cost to the employees to use the WMHP, which is a facilitating factor in seeking appropriate mental health care for employees.^41^ The reduction of this financial barrier may have resulted in more appropriate utilization of mental health services by WMHP users living with mental health conditions. Their return on investment (ie, better care at zero cost) was clearly positive. Whether this translated to a positive return on investment for their employers is still unknown.

Also, although nearly all claim totals and utilization metrics found lower health care claims cost and utilization rates for WMHP users than for comparison group members, the analyses performed here describe the experiences limited to 4 employer clients who allowed their data to be used in the study. This limits the generalizability of these findings to the populations described within this analysis. Future research should be conducted on the cost implications of WMHP use among employers in all industry categories.

In addition, the collection of detailed demographic information, such as race, ethnicity, and income, would facilitate future statements about the generalizability and applicability of the sample's findings. At the time of analysis, these data were not available.

Although the limitations described above are important, the results of this study still provide preliminary support for the hypothesis that using WMHP services for mental health concerns can be cost-effective, especially for individual users. With regard to quality clinical care, the services provided by Lyra's WMHP focus on EBT, resulting in superior rates of clinical symptom improvement and recovery not demonstrated by other solutions.^28,29^ Future research should evaluate the net cost of WMHP services to many more employers, to help inform decision making and enhance generalizability of the findings. Future research might also address the impact of WMHP providing EBT on employee productivity and turnover.

Many workforce mental health solutions now also involve a suite of services targeted at supporting mental health wellness,^42^ from prevention to treatment. Future research should expand the scope of evaluation to additional services offered through these programs and investigate potential savings and return on investment. In conclusion, this study provides support for the statement that EBT through a WMHP for mental health care may reduce overall medical insurance spending for those who make use of these services.

## Appendix

**Appendix Table A1.** Mapping from Diagnostic Impression to ICD-10 Diagnosis Codes

Lyra impression	ICD-10	ICD-10 description	CCS categories
Anger management	f4320	Adjustment disorder, unspecified	Adjustment disorders
Behavioral problems	f4320	Adjustment disorder, unspecified	Adjustment disorders
Career issues	f4320	Adjustment disorder, unspecified	Adjustment disorders
Communication problems	f4320	Adjustment disorder, unspecified	Adjustment disorders
Divorce	f4320	Adjustment disorder, unspecified	Adjustment disorders
Family conflict	f4320	Adjustment disorder, unspecified	Adjustment disorders
Grief	f4320	Adjustment disorder, unspecified	Adjustment disorders
Life transition	f4320	Adjustment disorder, unspecified	Adjustment disorders
Life purpose	f4320	Adjustment disorder, unspecified	Adjustment disorders
Other	f4320	Adjustment disorder, unspecified	Adjustment disorders
Parenting	f4320	Adjustment disorder, unspecified	Adjustment disorders
Parenting child behavior issues	f4320	Adjustment disorder, unspecified	Adjustment disorders
Relationship issues	f4320	Adjustment disorder, unspecified	Adjustment disorders
Self-esteem	f4320	Adjustment disorder, unspecified	Adjustment disorders
Substance abuse	f1010	Alcohol abuse, uncomplicated	Alcohol-related disorders
Anxiety	f419	Anxiety disorder, unspecified	Anxiety disorders
OCD	f429	Obsessive-compulsive disorder, unspecified	Anxiety disorders
Panic	f419	Anxiety disorder, unspecified	Anxiety disorders
Stress	f419	Anxiety disorder, unspecified	Anxiety disorders
Trauma	f4310	Post-traumatic stress disorder, unspecified	Anxiety disorders
ADHD	f909	Attention-deficit hyperactivity disorder, unspecified type	Attention-deficit conduct and disruptive behavior disorders Mental
Bipolar disorder	f319	Bipolar disorder, unspecified	Mood disorders
Depression sadness	f329	Major depressive disorder, single episode, unspecified	Mood disorders

ADHD, attention deficit hyperactivity disorder; CCS, Clinical Classifications Software; OCD, obsessive compulsive disorder.
